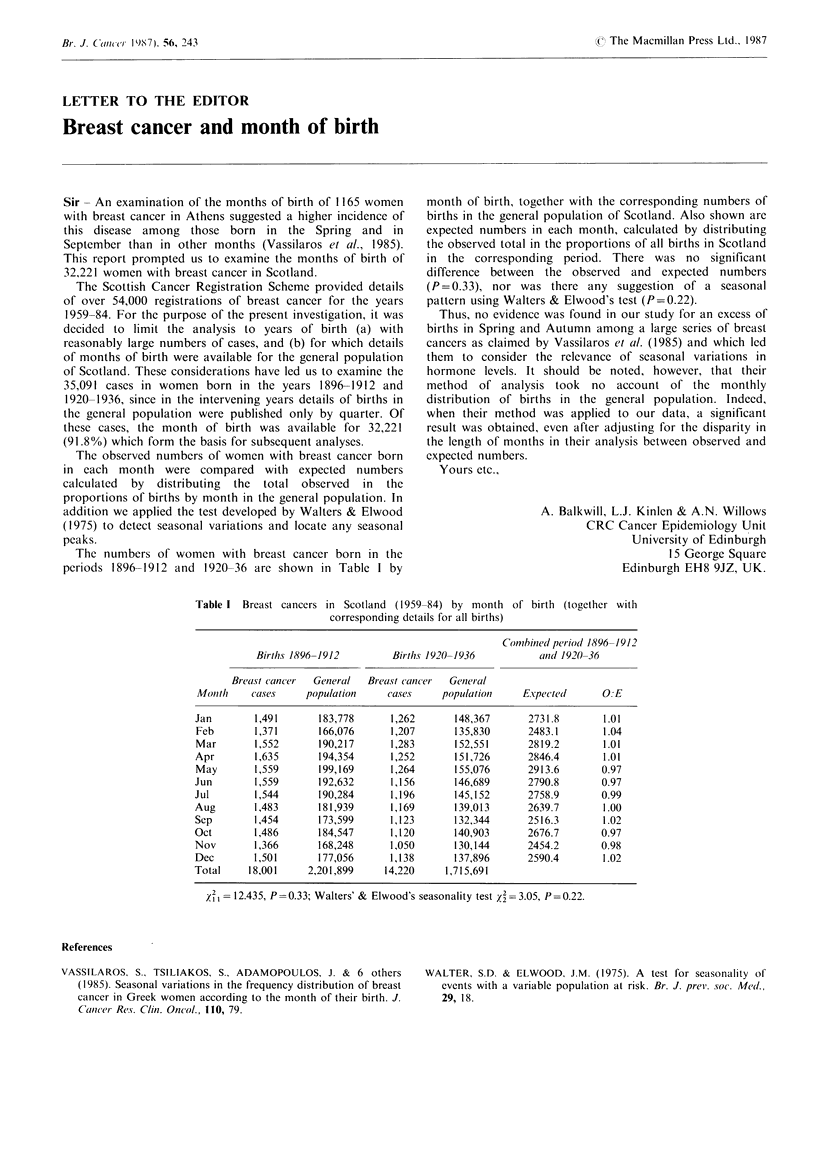# Breast cancer and month of birth.

**DOI:** 10.1038/bjc.1987.183

**Published:** 1987-08

**Authors:** A. Balkwill, L. J. Kinlen, A. N. Willows


 © The Macmillan Press Ltd., 1987

### LETTER TO THE EDITOR

### Breast cancer and month of birth

**Sir** – An examination of the months of birth of 1165 women with breast cancer in Athens suggested a higher incidence of this disease among those born in the Spring and in September than in other months (Vassilaros *et al.*, 1985). This report prompted us to examine the months of birth of 32,221 women with breast cancer in Scotland.

The Scottish Cancer Registration Scheme provided details of over 54,000 registrations of breast cancer for the years 1959–84. For the purpose of the present investigation, it was decided to limit the analysis to years of birth (a) with reasonably large numbers of cases, and (b) for which details of months of birth were available for the general population of Scotland. These considerations have led us to examine the 35,091 cases in women born in the years 1896–1912 and 1920–1936, since in the intervening years details of births in the general population were published only by quarter. Of these cases, the month of birth was available for 32,221 (91.8%) which form the basis for subsequent analyses.

The observed numbers of women with breast cancer born in each month were compared with expected numbers calculated by distributing the total observed in the proportions of births by month in the general population. In addition we applied the test developed by Walters & Elwood (1975) to detect seasonal variations and locate any seasonal peaks.

The numbers of women with breast cancer born in the periods 1896–1912 and 1920–36 are shown in Table I by month of birth, together with the corresponding numbers of births in the general population of Scotland. Also shown are expected numbers in each month, calculated by distributing the observed total in the proportions of all births in Scotland in the corresponding period. There was no significant difference between the observed and expected numbers ($P=0.33$), nor was there any suggestion of a seasonal pattern using Walters & Elwood's test ($P=0.22$).

Thus, no evidence was found in our study for an excess of births in Spring and Autumn among a large series of breast cancers as claimed by Vassilaros *et al.* (1985) and which led them to consider the relevance of seasonal variations in hormone levels. It should be noted, however, that their method of analysis took no account of the monthly distribution of births in the general population. Indeed, when their method was applied to our data, a significant result was obtained, even after adjusting for the disparity in the length of months in their analysis between observed and expected numbers.

Yours etc.,

A. Balkwill, L.J. Kinlen & A.N. Willows
CRC Cancer Epidemiology Unit
University of Edinburgh
15 George Square
Edinburgh EH8 9JZ, UK.

**Table I** Breast cancers in Scotland (1959–84) by month of birth (together with corresponding details for all births)

| | *Births 1896–1912* | | *Births 1920–1936* | | *Combined period 1896–1912 and 1920–36* | |
|---|---|---|---|---|---|---|
| *Month* | *Breast cancer cases* | *General population* | *Breast cancer cases* | *General population* | *Expected* | *O:E* |
| Jan | 1,491 | 183,778 | 1,262 | 148,367 | 2731.8 | 1.01 |
| Feb | 1,371 | 166,076 | 1,207 | 135,830 | 2483.1 | 1.04 |
| Mar | 1,552 | 190,217 | 1,283 | 152,551 | 2819.2 | 1.01 |
| Apr | 1,635 | 194,354 | 1,252 | 151,726 | 2846.4 | 1.01 |
| May | 1,559 | 199,169 | 1,264 | 155,076 | 2913.6 | 0.97 |
| Jun | 1,559 | 192,632 | 1,156 | 146,689 | 2790.8 | 0.97 |
| Jul | 1,544 | 190,284 | 1,196 | 145,152 | 2758.9 | 0.99 |
| Aug | 1,483 | 181,939 | 1,169 | 139,013 | 2639.7 | 1.00 |
| Sep | 1,454 | 173,599 | 1,123 | 132,344 | 2516.3 | 1.02 |
| Oct | 1,486 | 184,547 | 1,120 | 140,903 | 2676.7 | 0.97 |
| Nov | 1,366 | 168,248 | 1,050 | 130,144 | 2454.2 | 0.98 |
| Dec | 1,501 | 177,056 | 1,138 | 137,896 | 2590.4 | 1.02 |
| Total | 18,001 | 2,201,899 | 14,220 | 1,715,691 | | |

$\chi^2_{11}=12.435$, $P=0.33$; Walters' & Elwood's seasonality test $\chi^2_2=3.05$, $P=0.22$.

**References**

VASSILAROS, S., TSILIAKOS, S., ADAMOPOULOS, J. & 6 others (1985). Seasonal variations in the frequency distribution of breast cancer in Greek women according to the month of their birth. *J. Cancer Res. Clin. Oncol.*, **110**, 79.

WALTER, S.D. & ELWOOD, J.M. (1975). A test for seasonality of events with a variable population at risk. *Br. J. prev. soc. Med.*, **29**, 18.